# Accelerated Aging Effect on the Stability of the 3D-Printed Biodegradable Implant for Bone Defect Repairs

**DOI:** 10.3390/ma17246218

**Published:** 2024-12-19

**Authors:** Agnieszka Gutowska, Paweł Kubiak, Katarzyna Kośla, Bożena Wilbik-Hałgas, Edyta Chmal-Fudali, Agnieszka Kucharska-Jastrząbek, Marcin Henryk Struszczyk

**Affiliations:** Institute of Security Technologies “MORATEX”, 3 Marii Sklodowskiej—Curie Str., 90-505 Lodz, Poland; agutowska@moratex.eu (A.G.); kkosla@moratex.eu (K.K.); bhalgas@moratex.eu (B.W.-H.); efudali@moratex.eu (E.C.-F.); ajastrzabek@moratex.eu (A.K.-J.); mstruszczyk@moratex.eu (M.H.S.)

**Keywords:** accelerated aging, biodegradable implant, polylactide, nanohydroxyapatite, sterilization, 3D printing

## Abstract

This article presents an evaluation of the accelerated aging impact on the structural properties of biodegradable PLA/HAp implants produced using 3D printing technology for use in traumatic bone defect repairs in individual patients. The designed biodegradable implants were sterilized with a radiation dose of 25 ± 0.99% kGy, then exposed to an accelerated aging process. Selected physicomechanical and chemical properties of biodegradable implants were evaluated with FT-IR spectra analyses and DSC. The accelerated aging process, carried out according to the ASTM F 1980:2002 “Standard Guide for Accelerated Aging of Sterile Barrier Systems and Medical Devices”, simulates three years of implant usage. It confirmed the stability of structural, physical and mechanical properties and proved the effectiveness and safety of the implants’ application. The present study was conducted to determine the shelf-life of newly developed biodegradable implants proposed for the treatment of children and adolescents where bone growth still occurs by using accelerated aging methodologies, allowing the assessment of changes in performance that do not result in a negative impact on the safety of the medical device.

## 1. Introduction

The evolution of medicine is associated with a constant search for new solutions to improve patients’ health quality. In the search for new materials, the aim is to obtain biomedical materials with even better biocompatibility and tolerance of the immune system. Researchers are interested in materials that, through their specific properties, are able to fully integrate into the tissues of the body or be spontaneously removed from it when their function is fulfilled, as evidenced by the number of available scientific publications and inventions [1]. Biomaterials, which are synthetic or natural substances that come into contact with body tissues, can be used to replace entire or partial individual tissues and organs. It would be remiss of us not to mention the many different types of biomaterials that are currently in use, including metals, ceramics, polymers, carbon materials and composites. However, it should be noted that polymers and composites are the most biocompatible materials in tissue and bodily fluid environments. While some of these materials are capable of degradation over time after implantation, this is not a universal property. In addition to bio tolerance, materials for intra-body applications should also possess the required mechanical and technological properties. Knowledge of the above-mentioned requirements becomes the basis for considering the possibilities of selecting polymeric materials for manufacturing various types of short- and long-term implants [2,3].

One of the polymer materials that has found application in medical settings is poly(lactic acid)—PLA, also known as poly lactide (PLA). It is an aliphatic thermoplastic biodegradable polyester that occurs in two isomeric forms: (L) and (D). Synthetically produced lactic acid is a racemic variant, displaying equal quantities of the levo and dextro isomers [4,5,6]. The shelf-life stability of the biomaterials’ structural properties during storage is a main feature affecting clinical safety and performance. The unwanted changes in structural behavior of medical devices may change in the use risk (increase in risk effect or appearing of new risks) resulting in the loss of safety and performance. The chemical contamination, degradation, unsterile conditions and the unforeseen, significant alterations in the behavior of medical devices during the whole shelf-life are the main risks for additional assessment before implementation.

Moreover, the newly generated knowledge considering the behavior of material systems and/or the final medical device provides an explanation of the instability or stability of the medical devices resulting in outputs for the improvement in new design processes. Knowledge in the macro- and micro-structures changes, hence their dynamics during natural aging and the definition of the occurring range of changes allows design improvements [7].

In the literature, there is almost an absence of publications describing the phenomenon of the time-stability effect on the performance of the medical devices designed. In 2017, Khaja Moiduddin et al. described the preliminary research for the structural and mechanical behavior of personalized implants for cranioplasty made by additive manufacturing processes [8]. The Ti6Al4V ELI mesh implants developed in this study were characterized by high bone ingrowth permeability due to reduced weight and elasticity modulus closer to natural bone, which reduces the shielding effect against stress, but unfortunately, they were not biodegradable, which allows them to be used in a limited way for children and adolescents where bone growth still occurs.

Dong Hyeon Lee et. al. [9] summarize the methods for evaluating the long-term reliability of new implant designs based on thin polymer films in terms of the aspects of the metallic and polymeric packaging system application. The accelerated test theory was described in detail indicating the limitation and performance verification methods. As a key aspect of the accelerated testing, the increase in temperature was a limitation leading to the risk of additional polymer degradation and deterioration if the temperature of the body as the reference is used. The research carried out in this article was based on an in-depth analysis of the properties of both the packaging system and the implant itself, so that the selection of accelerated aging conditions took into account the elimination of the excess influence of temperature on the degradation of polymers from which both the packaging and the implant itself were designed.

Currently the essential requirements covered by the Medical Device Regulation (MDR) 2017/745 demand the stability confirmation of the medical devices, assumed key features for shelf-life (storage) estimated by the manufacturer. It is the most important attribute for the validation of medical device safety and performance. The consistency of the stability verification necessary during storage in real conditions is a significant element of designed biomaterials. There are currently no standard documents describing the appropriate method to estimate the time stability of designed medical devices. ASTM 1980 F Standard [10] guides the methodology for accelerated studies of packaging systems for medical devices using the Arrhenius equation [11].

In 2008 David, W. L. Hukins et al. [12] published theoretical research to better determine and understand the influence of temperature increase on the aging process for polymeric biomaterials and the medical devices proposed. In 2019, Jakob Janting et al. [13] conducted extensive research describing polymeric medical device aging and defined guidelines, based on averaging the Arrhenius reaction ratios if aging reference temperatures are higher than 25 °C. Therefore, to calculate the accelerated aging period for tested biodegradable implants, the standard Arrhenius equation was used. It was justified by the differences in effects originated from in vivo aging and the in vitro test. The aging system consisting of buffered physiological saline solution did not offer all the chemical and biological interactions that occurred in real conditions. Biodegradable implants were manufactured for the purpose of helping individual patients with injuries resulting in bone tissue loss. Such implants were characterized by a custom-designed shape intended to fill the defects that emerged in patients, and each implant was fabricated in a unique and individual shape based on the needs of the target patient. The accelerated aging was performed to evaluate the stability of their physical dimensions, mechanical and chemical properties while awaiting surgery. The implants are also biodegradable, meaning that they should decompose in the tissue environment simulating further growth, so there was no need to study the accelerated aging process for a longer period of time. Each time an implant was made, it should have immediately been given to treat the intended patient. Biodegradable 3D-printed implants intended for neurosurgical treatment were assessed for the accelerated aging effect on stability.

The general object of the performed research was to develop and verify the properties of biodegradable implants aimed at children and adolescents, where bone tissue is remodeled and grows, in which case the use of non-biodegradable implants is debatable. The aim of the study was to estimate the shelf-life of newly developed biodegradable implants by using accelerated aging methodologies, allowing the assessment of changes in functional properties that do not result in a negative impact on the safety of the medical device.

## 2. Materials

### 2.1. Biodegradable Implant

The composite used to design the 3D-printed implant was made of filament consisting of 90% polylactide (PLA) and 10% hydroxyapatite as a mineral additive. L-lactide and DL-lactide copolymer (80/20 molar ratio) with an inherent viscosity of 5.8 dL/g for the fabrication of the filament was applied [14,15].

**Figure 1:**
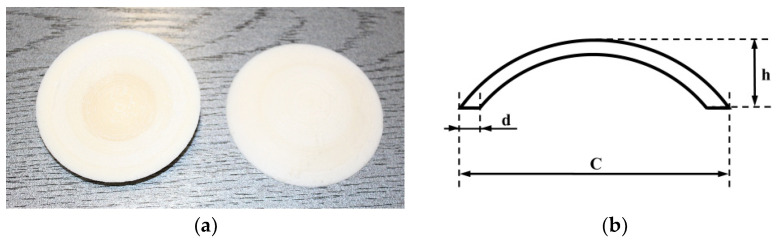
Implant model made by 3D printing from PLDLA filament: (**a**) sphere model (top and bottom view), (**b**) implant dimensions.

In this study, a sterilization process of the designed implant was carried out at 135 °C at a transportation velocity of 0.459 m/min ± 1.12% (electron beam “Elektronika”, Warsaw, Poland, accelerator with an energy of 10 MeV and an average power of 10 kW) using 25 ± 0.99% kGy irradiation dose (Institute of Nuclear Chemistry and Technology, Warsaw, Poland). The PLDLA implant model (in the shape of a sphere slice model) is shown in Figure 1 with its dimensions (diameter C = 70 mm, thickness d = 3 ÷ 4 mm and spline height h = 10 mm) in Figure 1.

### 2.2. Packaging System

#### 2.2.1. Medical Blister

To develop a packaging system, a 0.7 mm-thick blister produced from PET-G Type 200 polymer (Folienwerk Wolfen GmbH, Bitterfeld-Wolfen, Germany) was used (Poli Sp. z o. o., Inowrocław, Poland).

#### 2.2.2. PET-G Film

The used polyester PET-G raw material film (PAK Toruń Sp. z o.o., Toruń, Poland) with a thickness of 55 µm for covering the packaging system has a heat-sealable film on the inner side (used on the inside of the packaging system).

The packaging system was produced by sealing the blister and PET-G film together using a MAGVAC 520 MED vacuum sealer (TECHNO P.P.H.U. S. j., Warsaw, Poland).

The following sealing parameters were used:

- Sealing temperature: 160 °C;
- Sealing time at this stage was set in 3 variants: 1 s, 1.5 s, 2 s and the seal parameters were verified;
- Selecting the optimal sealing time;
- Air extraction while sealing the last edge: 0.1 s.

The process required cutting a sheet of PET-G film according to the dimensions of the blister and then sealing each side of the package with the film placed on top, with the sealing film on the blister side. While sealing the last edge, there was an additional vacuuming of air, which made the surface of the package smooth. For further tests to assess the effect of the studied accelerated aging on the behavior of the packaging system, the following welding parameters were selected:

- Sealing temperature: 160 °C;
- Sealing time: 2 s;
- Air extraction while sealing the last edge: 0.1 s.

**Figure 2:**
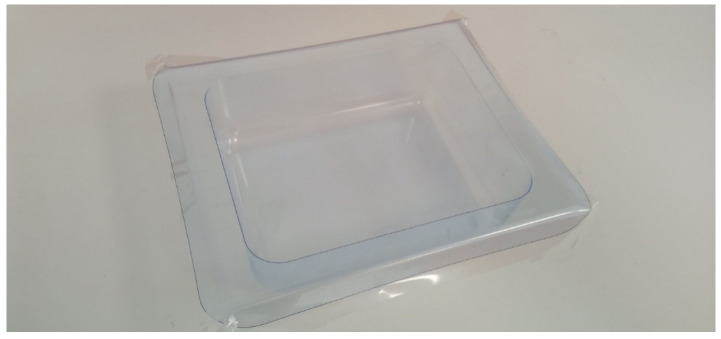
Packaging system prototype for implant.

A packaging system prototype built using the PET-G film and the medical blister is shown in Figure 2.

### 2.3. Methods

#### 2.3.1. Fourier-Transform Infrared Spectroscopy (FTIR)

FTIR spectrophotometer (Thermo Scientific Nicolet iS10, Waltham, MA, USA) was applied to characterize the functional groups of the composite material of the designed implant. The background and the spectrum of the investigated implant were determined so as to avoid the negative effects caused by the instrument and the environmental conditions. The average of three independent measurements per sample in the range of wavenumbers 4000 cm^−1^÷600 cm^−1^ were performed.

The following parameters were used during the FTIR study: measurement recording accuracy: 2; mirror speed: 0.31/s; aperture: 50; minimum recorded scans number: 32; and ITR (Thermo Scientific) reflective snap type with the diamond crystal of a reflection angle at 45°.

Table 1 shows the FTIR spectrophotometer operating parameters.

#### 2.3.2. Differential Scanning Calorimetry (DSC)

DSC measurements were carried out using a Mettler Toledo differential scanning DSC calorimeter (DSC 3 Mettler Toledo, Greifensee, Swizerland) calibrated using typical standards (indium, n-octane). The DSC test was performed in a nitrogen atmosphere using a standard 40 μL aluminum crucible. Liquid nitrogen was used as a cooling agent. 

The procedure was carried out by heating in two stages and cooling as follows:

- STEP I—heating 25 °C ÷ 200 °C with a rate of 10 °C/min and gas flow of 60 mL/min;
- STEP II—cooling 200 °C ÷ 25 °C with a rate of 10 °C/min and gas flow of 60 mL/min;
- STEP III—heating 25 °C ÷ 500 °C with a rate 10 °C/min and gas flow of 60 mL/min.

#### 2.3.3. Thickness Determination

To determine the average thickness, 5 samples were prepared in the form of a spherical surface shown on Figure 1.

The samples were acclimatized for 24 h in a normal climate in accordance with PN-EN ISO 139:2006 [16] and then the distance between the layers of samples in a spherical surface slice shape was measured using calipers. The unit thickness results were determined to the nearest 0.001 mm. The arithmetic mean of the thicknesses was given from 5 measurements expressing the final result in three significant digits in [mm].

#### 2.3.4. Apparent Density of Determination

To determine the apparent density, 5 samples were prepared in the form of a spherical surface shown in Figure 1.

The samples were acclimatized for 24 h in a normal climate [16] and then the surface area of each working sample was calculated to the nearest 1 mm^2^.

The apparent density ρ in g/m^3^ was calculated using the equation:

(1) $$\rho =\frac{m}{V}\times 10^{9}$$

where

*m*—sample mass [g];
*V*—sample volume [mm^3^].

The arithmetic average is given from 5 measurements, expressing the final result to the nearest 1 [g/m^3^].

#### 2.3.5. Areal Density Determination

To determine the areal density, 5 samples were prepared in the form of a spherical surface shown in Figure 1.

The samples were conditioned for 24 h in a normal climate [16] and then the surface area of each working sample was calculated by the following equation:

(2) $$m_{p}=\frac{m}{S}\times 10^{6}$$

where

*m*—sample mass [g];
*S—*sample surface area [mm^2^].

The arithmetic average is given from 5 measurements, expressing the final result to the nearest 1 [g/m^2^].

#### 2.3.6. Rounding Height of a Sphere Section Determination

To determine the rounding height of a sphere, samples were prepared in the form of a spherical surface shown in Figure 1.

The specimens were conditioned for 24 h in a normal climate [16] and then the height of the implant splay was measured using a thickness gauge to the nearest 0.01 mm.

The arithmetic mean was given from 5 measurements, expressing the final result in three significant digits in [mm].

#### 2.3.7. Maximum Compression Force Determination

To determine the maximum compression force, 5 samples were prepared in the form of a spherical surface shown in Figure 1.

The specimens were conditioned for 24 h in a normal climate [16]. The implant splay height and thickness were determined for each test sample. The test sample was placed on the lower plate of the compression apparatus (Zwick/Roell Type 1456 RetroLine, Ulm, Germany), so that its center point coincided with the axis of the machine. Then, the apparatus was set at the zero point so that the top plate touched the prosthesis. The compression test was performed at 1 mm/min until the specimen was destroyed or the distance between the plates was equal to the thickness of the specimen—completely flattening the prosthesis.

While performing the test, the following were determined: maximum force at compression, deflection at maximum force, and force at 1 mm, 2 mm and 3 mm deflection.

This test determined the force corresponding to the deflection by measuring up to 4.5 mm of the implant by the percentage of the strain in relation to the internal height (10 mm) of the implant proving its safety for internal usage.

#### 2.3.8. Deflection at 2, 3 and 4 mm and at Maximum Force Determination

To determine the deflection at 2, 3 and 4 mm and at maximum force, 5 samples in the form are shown in Figure 1.

The specimens were conditioned for 24 h in a normal climate [16]. The thickness and width were determined for each unit test sample. The test sample was placed in the clamps. The longitudinal axis corresponded completely with the machine axis (Zwick/Roell Type 1456 RetroLine, Germany). The specimen was stretched along its major axis at a constant speed of 1 mm/min until the specimen failed (broke). While the test was being conducted, the maximum tensile force, maximum force elongation, tensile failure force, elongation at failure force and tensile modulus were determined.

#### 2.3.9. Accelerated Aging

The accelerated aging tests were carried out based on the guidelines of ASTM F 1980:2002 [10]. The aging simulation included incubation samples of implants placed in the packing system and tested in a climatic chamber at a humidity of (50 ± 2)%, a temperature of (65 ± 2) °C and a specified time interval of 56 days, corresponding to a simulated aging time of 3 years’ storage (implant samples intended for sterility, thermal and structural testing). The accelerated aging was simulated by applying the Arrhenius equation [10]:

(3) $$AAF=Q_{10}\left[\frac{T_{AA}-T_{RT}}{10}\right]$$

where

*AAF*—accelerated aging factor;
*T_AA_—*accelerated aging temperature [°C];
*T_RT_—*reference temperature [°C];
*Q*_10_—aging coefficient originated from the alteration in kinetics in tested parameters with an increase in temperature by 10 °C [10].

The accelerated aging time was estimated based on the below formula [10]:

(4) $$ATT=\frac{356\;days}{AAF}$$

where

*ATT*—accelerated aging time [10].

T_AA_ is the main factor influencing the duration of the accelerated aging. According to ASTM 1980F:2002 [10], the T_AA_ range ought to be 50 °C ÷ 65 °C. Higher temperatures clearly shorten the aging process. In other words, T_AA_ can negatively influence the structural behavior of samples resulting in differences in originated results in relation to the aging in real conditions. The *Q*_10_ parameter, which determines the aging curve, was set to the default value of 2, and an ambient temperature of 22 °C was assumed.

## 3. Results and Discussion

### 3.1. FTIR Study

**Figure 3:**
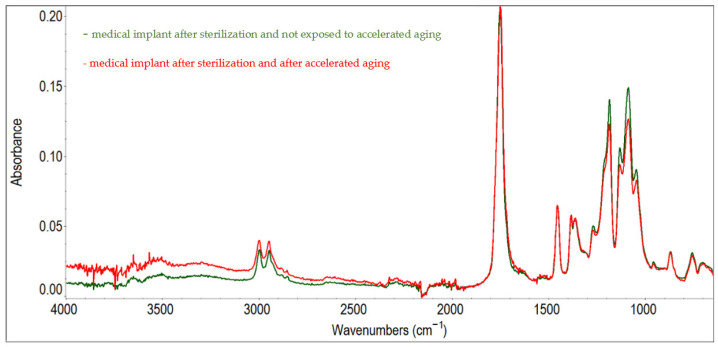
FT-IR spectra of an implant after radiation sterilization with a dose of untreated 25 kGy and subjected to accelerated aging, simulating 3 years of storage in real conditions.

In order to guarantee the precision and dependability of the research findings, the implant FTIR spectrum underwent three rounds of measurements. The FTIR spectrum was estimated by the ratio between the sample and the background spectra. The sample FTIR spectrum was divided by the background spectrum (a process known as “rationing”) to eliminate the potential impact of the instrument and atmospheric conditions on the results. This resulted in a final FTIR spectrum that was clear from any signals that may have been present as a result of these external factors, and which displayed only those signals that originated from the sample itself. Following the completion of the measurements, the results were averaged, and automatic baseline corrections were performed. The results of the FTIR study are shown in the plots of spectra with the sample-specific peaks highlighted (Figure 3).

The following values of wavenumbers were used to obtain the characteristic peaks at *λ* = 755 cm^−1^, *λ* = 867 cm ^−1^, *λ* = 1045 cm^−1^, *λ* = 1086 cm^−1^, *λ* = 1129 cm^−1^, *λ* = 1183 cm^−1^, *λ* = 1382 cm^−1^, *λ* = 1453 cm^−1^, *λ* = 1747 cm^−1^, *λ* = 2946 cm^−1^ and *λ* = 2995 cm^−1^. The most intense bands in the range of 1300 ÷ 1000 cm^−1^ and at *λ* = 1748 cm^−1^ are associated with tensile vibrations, based on the bonds ν(C-O) and ν(C=O). Tensile and bending vibrations of methyl groups -CH_3_ are also visible in the shape of bands that are present in ranges of 3100 ÷ 2800 cm^−1^ and 1480–1300 cm^−1^. Bands present at *λ* = 867 cm^−1^ and *λ* = 755 cm^−1^ represent ν(C-C) bond vibrations. The recorded FTIR spectrum for the polymer corresponds with the literature data [17,18].

After an accelerated aging of the designed implant equal to storage by 3 years, no alteration in the bands toward higher or lower values of the wavenumbers were observed. However, a significant decrease in the bands’ intensity located in the range of wavenumbers 755 ÷ 1747 cm^−1^ was observed. The changes in the area under the bands may suggest degradation of the PLDLA. The FTIR spectrum of accelerated aging implant indicated that the groups most sensitive for degradation are C=O carbonyl and C-O-C. The accelerated aging effect on the structure of the PLDLA polymer was observed and conformed in the FTIR spectrum of the aged implants in accelerated conditions.

### 3.2. DSC Study

**Figure 4:**
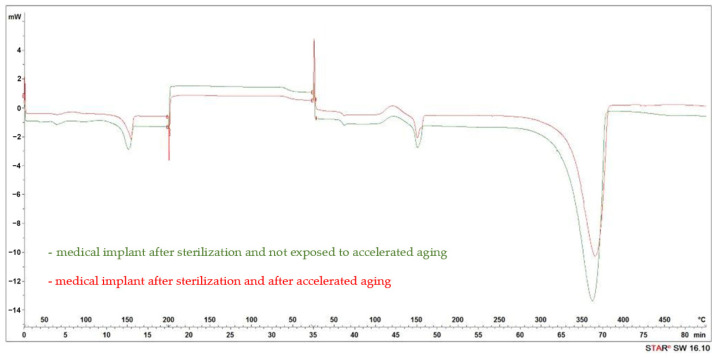
DSC curve of the implant after radiation sterilization at a dose of 25 kGy, unaged and subjected to accelerated aging, simulating 3 years of storage in real conditions.

On the basis of the achieved DSC thermograms, the range of temperatures and the heating effect (enthalpy) of the crystallization and melting processes of the crystalline phase were determined. Two endothermic peaks were recorded as a result of heating a 3D-printed implant sample not subjected to accelerated aging, with a maximum transition temperature between 64 °C and 151 °C (Figure 4).

No changes in the DSC waveform during the cooling process was found in the form of peaks in the expected temperature ranges. This phenomenon is related to the crystallization process, while a slightly outlined spike occurred at the temperature of about 60 °C, characteristically for glazing (Figure 4). When reheating the tested implant, the transition associated with the glazing at a temperature of approximately 60 °C was recorded, followed by an exothermic peak associated with the crystallization process, the maximum of which was recorded at a temperature of 121 °C. Upon completion of the crystallization process, the melting of the crystalline phase was observed, resulting in an endothermic peak with a maximum transformation temperature of 151 °C. A notable endothermic peak, with a maximum transition temperature of approximately 370 °C, was identified and is believed to be associated with thermal degradation.

The accelerated aging of the 3D-printed and designed implant did not affect the nature of the thermal waveforms and the maximum values of the recorded transformations. However, the enthalpy values of the transformations changed, reflecting in the crystallinity index (Xc) calculated after the first and second heating (XcII). The accelerated aging yield increased the crystallinity index (Xc) from 33% to 39%, whereas the crystallinity index after the second heating (XcII) rose from 24% to 34%.

### 3.3. Physicomechanical Studies Results

**Figure 5:**
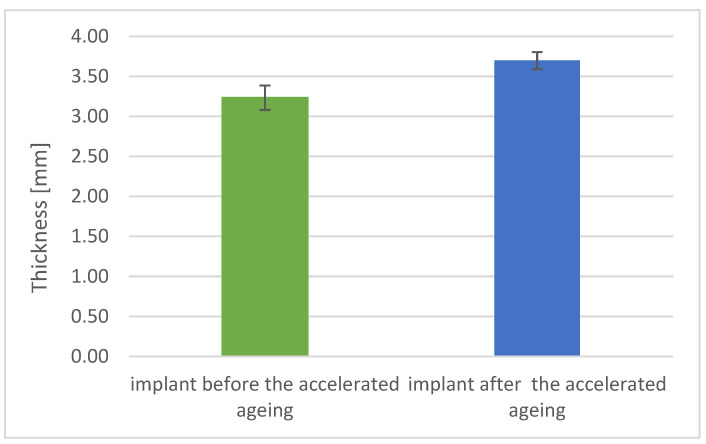
Thickness of implant before and after accelerated aging.

**Figure 6:**
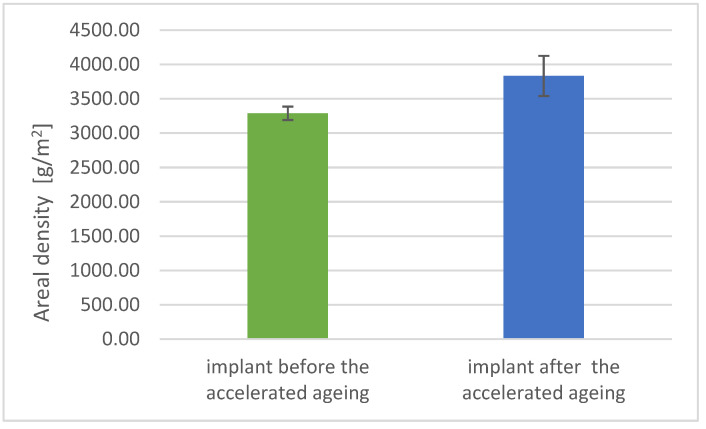
Areal density of implant samples before and after accelerated aging.

**Figure 7:**
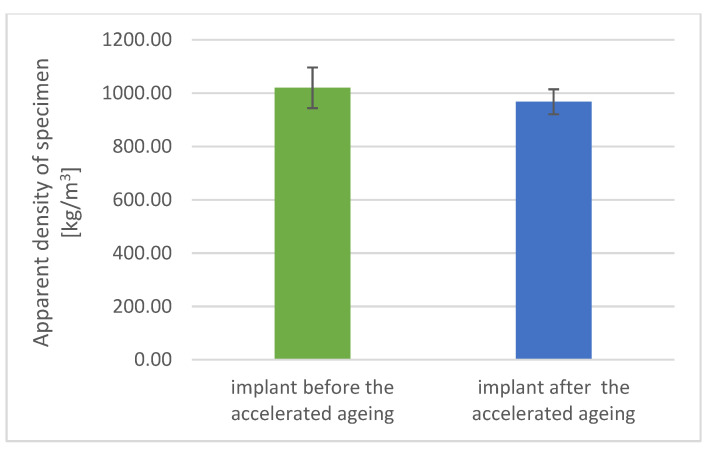
Apparent density of implant before and after accelerated aging.

**Figure 8:**
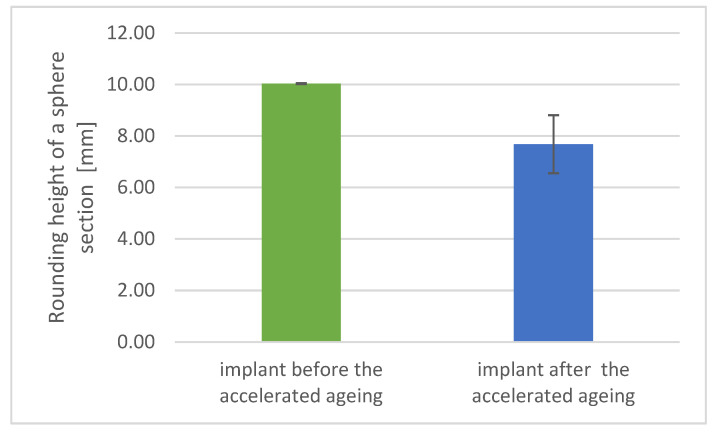
Rounding height of implant before and after accelerated aging.

For implant models in the shape of a 3D sphere fragment, after radiation sterilization with a dose of 25 kGy, subjected and not subjected to accelerated aging process (equals 3 years of storage in real conditions), tests were performed for the evaluation of physical and mechanical properties. The details of test results are presented below (Figure 5, Figure 6, Figure 7 and Figure 8). The thickness of spherical implants increased after accelerated aging by an average of 14% compared to the thickness of implants before this process (Figure 5).

The alteration in the tested parameters corresponds to the change in areal density (increase by 17% on average, Figure 7) and a decrease in the apparent density of samples aged by acceleration (by an average of 5%, Figure 8).

Significant changes can be observed in the rounding height of the implants (Figure 8) which decreased by 25%. The observed change of the rounding height after the accelerated aging process directly proves the assumed and planned degradation of the implant in time after the procedure.

It also proves the implant suitability for internal use and proves the basic therapeutic features of the product, which include, among other things, the ability to accelerate the regeneration processes of bone defects. The above-observed changes do not significantly affect the physicomechanical properties in the same way that they affect the safety and performance of the evaluated implant.

**Figure 9:**
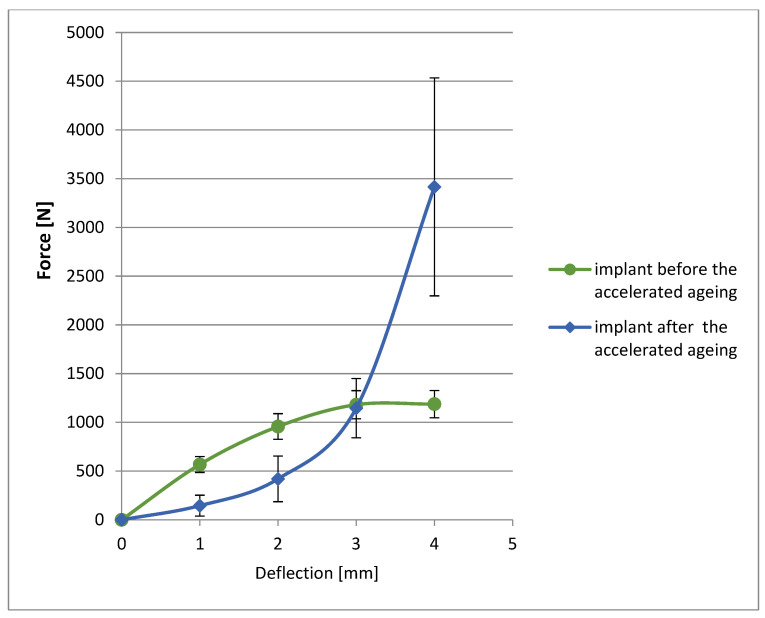
Deflection for spherical surface implant before and after accelerated aging.

The results of the compressive mechanical properties in the spherical surface implant samples before and after accelerated aging are shown in Figure 9.

The most important physicomechanical properties of 3D-printed implants are summarized in Table 2.

Significant changes in deflection characteristics can be observed in the spherical surface implant samples before and after aging. Much more force is needed at the beginning of deflection for the implant not exposed to aging at accelerated conditions, while the implant requires less force to deflect after accelerated aging. It proves the intended degradation in the structure of the implant and the reduction of its brittleness because this implant was designed to stimulate bone tissue to grow rapidly and was intended to provide a frame that would biodegrade over the treatment period.

The tested implant samples were characterized by a three-dimensional porous structure similar to the natural properties of bones, reproducing their porous form. The apparent density of the implant in the form of a spherical surface before the aging process was detected in 1020 ± 76 kg/m^3^ and in 968 ± 47 kg/m^3^ after the aging process.

Differences in the areal density of the implants after the accelerated aging process, corresponding to 3 years of use and/or storage, were observed, ranging from 3.5% to about 13% and from 3% to 32%, respectively. For apparent density, the difference in the obtained values equals 7÷9%.

In [19], the wide range of the research on biodegradable implants based on the PLA/HAp composition development was described. Also, the verification of the mechanical and rheological properties of the implant was performed. However, the verification of the change in the mechanical and physical behavior, even in accelerated conditions, was not investigated. Moreover, the changes in mechanical and physical properties that occur during accelerated time or natural aging under normal conditions have not been studied. Understanding the aging process (aging degradation) is important. The recognition of the medical device behavior changes during aging, resulting in estimating their shelf-life limits for safe use, and this is most important especially for biodegradable products.

After accelerated aging, the PLDLA models of implants fabricated by 3D printing maintained the tested mechanical properties and porosity of the structure, despite being made of bioresorbable materials. The tensile modulus did not change significantly; the maximum tensile force remains at a similar level. Also, the initial tensile stress values (before aging) did not significantly change after aging, which is expected and intended by the implant application.

## 4. Conclusions

The aim of the present research was focused on determining the impact of accelerated aging on the structural characteristics of a developed implant produced by 3D printing and subjected to radiation sterilization with the assumed application for children and adolescents where bone growth still occurs. The biodegradable implant was made using biodegradable PLA polymers. Thus, no further biodegradation studies were required. This implant was designed to stimulate rapid bone tissue growth and provides a biodegradable frame over the course of the treatment. Selected physicomechanical properties, which are essential from the clinical usability perspective of the developed biodegradable medical devices subjected to accelerated aging, were assessed. The research on accelerated aging was conducted by taking into account procedures based on Polish and European normative documents, including the guidelines of the standard.

Major changes in deflection characteristics for the spherical surface implant can be found after aging. At the beginning of the deflection in the implant not subjected to accelerated aging, it is clearly seen that much more force is needed, while after the accelerated aging process, the implant requires less force to deflect. This confirms the anticipated degradation of the implant structure and reduction in brittleness, proving its effectiveness and biodegradable role in medical application. The observed change in the implant rounding height following the accelerated aging process verifies the anticipated degradation over treatment. Also, the structural degradation of the PLDLA polymer was observed in the FTIR analysis. This validates the suitability of the implant for internal use and confirms the fundamental therapeutic capabilities including the ability to accelerate bone defect regeneration. The conducted research has shown that the storage conditions of biodegradable implants are important in terms of maintaining their functional properties affecting safety. The mileage has changed the deflection in the spherical surface implant samples corresponding to the alteration in physicomechanical and structural behavior.

The next stage of the research will be the validation of observations made from studies of aging and in vitro degradation as part of in vivo studies.

## Figures and Tables

**Table 1:** Operating parameters of the device.

Measurement Parameters	Value	Recommended Wavenumber Range	4000 ÷ 650 cm^−1^
Number of scans	32	Amplification	2.0
Resolution	4	Velocity	0.4747
Correction	ATR	Tightness	Medium Resolution
Automatic atmospheric correction	Enabled		
Optics Parameters	Value		
Sample range	Ex-display		
Detector	DTGS KBr		
Beam splitter	KBr		
Source	IR		
Appetizer	ATR		
Window	ZnSe		

**Table 2:** Summary of 3D-printed implant physical parameters before and after aging.

	Before Accelerated Aging Process		After Accelerated Aging Process	
3D-Printed Implant Parameter	Average Value	Standard Deviation (SD)	Average Value	Standard Deviation (SD)
Mass [g]	14.14	0.41	15.35	0.30
Thickness [mm]	3.23	0.15	3.70	0.11
Apparent Density [kg/m^3^]	1020	76	968	47
Areal Density [g/m^2^]	3289	97	3833	291
Rounding height [mm]	10.03	0.02	7.68	1.12

## Data Availability

The raw data supporting the conclusions of this article will be made available by the authors on request due to privacy/ethical.
